# AO Spine Clinical Practice Recommendations for the Management of Spinal Tuberculosis

**DOI:** 10.1177/21925682261474130

**Published:** 2026-07-30

**Authors:** Julian Scherer, Charlotte Dandurand, Vishal Kumar, Atiq Uz Zaman, Gaston Camino-Willhuber, Charles Fisher, Gregory D. Schroeder, Mohammad El-Sharkawi, Richard Bransford, Harvinder Singh Chhabra, Andrei F. Joaquim, Sebastian F. Bigdon

**Affiliations:** 1 General Medicine & Global Health (GMGH), Department of Medicine and Orthopaedic Research Unit (ORU), Division of Orthopaedic Surgery, Faculty of Health Science, 37716 University of Cape Town, Cape Town, South Africa; 2 Center for Musculoskeletal Surgery, 14903 Charité Universitätsmedizin Berlin, Berlin, Germany; 3 Combined Neurosurgical and Orthopedic Spine Program, Vancouver General Hospital, 8166 University of British Columbia, Vancouver, BC, Canada; 4 Additional Professor of Orthopaedics, 29751 PGIMER, Chandigarh, India; 5 Lahore Medical & Dental College, Orthopaedic & Spine Surgery Department, 602893 Ghurki Trust Teaching Hospital, Lahore, Pakistan; 6 Department of Orthopedic Surgery and Traumatology, Quirónsalud Group, 58379 Policlínica Gipuzkoa, San Sebastián, Spain; 7 Department of Orthopaedic Surgery, 387400 Rothman Institute at Thomas Jefferson University Hospital, Philadelphia, PA, USA; 8 Department of Orthopaedic and Trauma Surgery, Faculty of Medicine, 68796 Assiut University, Assiut, Egypt; 9 Department of Orthopaedic Surgery and Sports Medicine, University of Washington, 21618 Harborview Medical Center, Seattle, WA, USA; 10 309539 Department of Spine and Rehabilitation centre, Sri Balaji Action Medical Institute, New Delhi, India; 11 Neurosurgery Division, Department of Neurology, 28132 State University of Campinas, Campinas-Sao Paulo, Brazil; 12 Department of Traumatology and Orthopaedic Surgery, 27252 Inselspital University of Bern, Bern, Switzerland

**Keywords:** FDG-PET/CT, GRADE methodology, spinal tuberculosis, spine, spondylodiscitis

## Abstract

**Study design:**

Literature review with clinical recommendations.

**Objective:**

To highlight impactful studies on the management of spinal tuberculosis (STB), identified by the AO Spine Knowledge Forum Trauma and Infection group, with recommendations for their integration into clinical practice.

**Methods:**

Four studies addressing key phases of STB management — imaging diagnosis, laboratory diagnosis, surgical indication and approach, and imaging-based healed status — were reviewed. Consensus recommendations of the KF opinion leaders were graded as strong or conditional following the GRADE methodology.

**Results:**

Altini et al evaluated the diagnostic value of MRI and FDG-PET/CT in spondylodiscitis including STB; we conditionally recommend FDG-PET/CT as an adjunctive imaging modality when MRI is inconclusive, contraindicated, or limited by artifact. Hu et al developed a multivariable laboratory prediction model based on seven routine indices for early STB diagnosis, achieving an AUC of 0.947; we conditionally recommend this model as an adjunctive screening tool when definitive microbiological testing is delayed or unavailable. Tang et al compared anterior-only, posterior-only, and combined surgical approaches in thoracolumbar STB; we conditionally recommend a posterior-only approach as the preferred surgical strategy in most cases requiring operative treatment. Mittal et al evaluated FDG-PET/CT and contrast MRI for defining healed status and guiding treatment completion; we conditionally recommend FDG-PET/CT as an adjunctive tool when the treatment endpoint remains uncertain or MRI is limited by metallic implants.

**Conclusion:**

This review provides spine surgeons and clinicians with evidence-based, expert-graded recommendations to enhance standardization and effectiveness across the key clinical decision points in spinal tuberculosis management.

## Disclaimer for Special Review Articles

Please note that this article has been submitted to the special review article series routinely published by the Global Spine Journal and does not follow the standard article format. Instead, this article follows a unique format and methodology endorsed by our Deputy Editors and Editors-in-Chief. The authors select 3-4 articles on a specific topic. For each article they provide the clinical rationale, summarize the findings, and assess the quality of evidence using GRADE criteria. Based on the methodological quality and the expert opinion from a diverse panel of key opinion leaders, they formulate recommendations on whether the evidence should be integrated into clinical practice.

The purpose of this article series is to serve as a knowledge translation tool to help busy clinicians efficiently interpret evidence and apply it to make their practice and patients better.

## Introduction

Spinal tuberculosis (STB) remains one of the most consequential forms of extrapulmonary tuberculosis. Delayed or inadequate treatment may result in vertebral destruction, spinal deformity, neurologic compromise, chronic pain, and prolonged disability.^1,2^ Despite advances in imaging, microbiological testing, and surgical techniques, important aspects of STB management remain challenging in daily practice. In many patients, the diagnosis is not established immediately, imaging findings may be equivocal, access to definitive microbiological confirmation may be delayed, and the optimal timing and extent of intervention must often be determined before full diagnostic certainty is available.^3,4^

For the practicing spine surgeon, the most relevant questions are therefore not only whether STB is present, but how to proceed when the diagnosis is uncertain, which adjunctive tools may improve early decision-making, when a surgical approach should be favored over another, and how healed status should be defined at the end of treatment. These questions are particularly important because STB is encountered across highly variable health-care environments, ranging from resource-constrained settings with limited access to advanced diagnostics to tertiary referral centers with complex surgical and diagnostic practice.^5,6^

This article was developed to help translate selected evidence into clinically useful recommendations for surgeons treating patients with STB. Rather than providing a comprehensive review of all aspects of spinal tuberculosis, we selected four studies that address common decision points along the patient journey. The first evaluates the role of FDG-PET/CT as an adjunct when MRI is inconclusive, contraindicated, or limited by artifact. The second examines whether routinely available laboratory indices can support earlier diagnostic suspicion in patients with possible STB. The third focuses on surgical approach selection in thoracolumbar spinal tuberculosis once the indication for surgery has been established. The fourth addresses one of the most difficult unresolved problems in STB care: how to define imaging-based healed status and thereby reflect the end-point of antitubercular therapy.

For each article, we summarize the clinical rationale, key findings, methodological strengths and limitations, and then provide a recommendation for integrating the evidence into practice using GRADE-informed language. Our goal is not to overstate the certainty of the available literature, but to identify where the current evidence can reasonably inform decision-making now, while acknowledging the limitations that remain.

All authors independently reviewed each selected study. Evidence quality was assessed using the GRADE (Grading of Recommendations Assessment, Development and Evaluation) framework, considering risk of bias, indirectness, inconsistency, imprecision, and publication bias. Recommendations were classified as strong or conditional based on the certainty of evidence and the balance of benefits and harms. The panel consisted of the co-authors of this article, who are members of the AO Spine Knowledge Forum Trauma and Infection group, representing a diversity of geographic and clinical expertise in spinal tuberculosis management. Consensus was reached through structured group discussion, and no major disagreements requiring formal resolution arose.

### Imaging “Diagnosis” of Spinal Tuberculosis

Article 1: Altini C, Lavelli V, Niccoli-Asabella A, Sardaro A, Branca A, Santo G, et al. Comparison of the Diagnostic Value of MRI and Whole Body F-18-FDG PET/CT in Diagnosis of Spondylodiscitis. Journal of Clinical Medicine. 2020;9.

#### Clinical Rationale

Spinal tuberculosis is frequently diagnosed late because symptoms are nonspecific and microbiological confirmation is often delayed. MRI remains the primary imaging modality for suspected STB because it defines the extent of bony destruction, epidural involvement, deformity, and neural compression.^7^ However, MRI may be inconclusive, limited by artifact, or contraindicated in selected patients.^8^ The study by Altini et al was selected because it examines whether FDG-PET/CT can provide clinically useful adjunctive information when conventional imaging alone does not adequately answer the diagnostic question.^9^

#### Clinical Summary

Altini et al retrospectively evaluated 56 patients with suspected spondylodiscitis who underwent both MRI and whole-body FDG-PET/CT; 18 patients had STB. MRI demonstrated sensitivity of 100%, specificity of 60%, and accuracy of 97%, whereas FDG-PET/CT showed sensitivity of 92%, specificity of 100%, and accuracy of 94%. In the STB subgroup specifically (n=18), both modalities were concordant in 16 of 18 patients; the two discordant cases were false-negatives on FDG-PET/CT, likely attributable to low-virulence organisms or isolated epidural involvement. These findings suggest that MRI remains the preferred first-line imaging modality, whereas FDG-PET/CT may be most helpful when MRI findings are equivocal, technically limited, or contraindicated.

#### Methodological Review

This was a retrospective diagnostic study with a small sample size, which limits certainty. Its most important limitation is indirectness: the cohort included patients with spondylodiscitis broadly, and only a subset had spinal tuberculosis. Therefore, the findings cannot be interpreted as STB-specific diagnostic evidence. Nonetheless, the comparison is clinically relevant because both modalities were applied within the same cohort, allowing a pragmatic estimate of their relative strengths. Overall, the quality of evidence is **Low** due to retrospective design, small sample size, and indirectness.

#### Recommendation for Integrating Into Clinical Practice

**Based on GRADE methodology, we conditionally recommend FDG-PET/CT as an adjunctive imaging modality in selected patients with suspected spinal tuberculosis when MRI is inconclusive, contraindicated, or substantially limited by artifact.** MRI should remain the first-line imaging modality for suspected spinal TB. FDG-PET/CT may be particularly useful when additional metabolic information is needed to distinguish active infection from alternative causes of structural change, but its routine use is limited by cost, availability, and the low-certainty, indirect nature of the supporting evidence. FDG-PET/CT does not establish a diagnosis of spinal tuberculosis, cannot replace microbiological confirmation, and is not recommended as a routine investigation in resource-limited or endemic settings where cost and availability constrain its use.

### Laboratory “Diagnosis” of Spinal Tuberculosis

Article 2: Hu X, Zhang G, Zhang H, Tang M, Liu S, Tang B, et al. A predictive model for early clinical diagnosis of spinal tuberculosis based on conventional laboratory indices: A multicenter real-world study. Front Cell Infect Microbiol. 2023;13:1150632.

#### Clinical Rationale

Early diagnosis of spinal tuberculosis remains difficult because the presenting symptoms and imaging features often overlap with pyogenic spinal infection, brucellosis, and neoplastic disease. Definitive diagnosis usually depends on biopsy, culture, histopathology, or molecular testing, but these investigations may be invasive, delayed, or unavailable in routine practice.^4,10^ Hu et al selected a clinically important question: whether routinely available laboratory indices could be combined into a practical early diagnostic model to help identify patients with probable STB before confirmatory testing is obtained, particularly in resource-constrained settings.^11^

#### Clinical Summary

Hu et al performed a retrospective multicenter study including 206 patients with suspected spinal tuberculosis from four hospitals, of whom 105 had confirmed STB and 101 had non-STB diagnoses. The non-STB group included pyogenic spinal infection, brucellosis, tumors, fungal infection, and viral infection. Forty routine admission variables were screened, and a multivariable logistic model was developed using LASSO selection followed by regression modeling. Seven variables were retained in the final nomogram: TB antibody, IGRAs, RBC count, monocyte percentage, RDW, AST, and BUN. The nomogram is applied by locating each patient’s value for the seven variables on the respective axis, reading off the corresponding point score, summing the scores to a total, and reading the predicted probability of STB from the total points scale. A total score above approximately 400 points corresponds to a predicted probability exceeding 0.5, indicating likely STB. All seven variables are obtainable from routine admission blood work, making the tool accessible without additional investigations. The model showed high discrimination, with an AUC of 0.9468 in the training cohort and 0.9188 in the validation cohort. Importantly, most single laboratory markers performed poorly in isolation, whereas the combined model substantially improved diagnostic discrimination. The study therefore suggests that routine laboratory and immunologic indices may help raise suspicion for STB early in the diagnostic pathway, especially when definitive microbiological testing is not immediately available.

#### Methodological Review

This study has several strengths, including a multicenter design, inclusion of clinically relevant comparison diagnoses, and use of routine admission tests that are widely available. The modeling strategy was appropriate, with variable screening by LASSO regression and internal validation in a separate subset of the dataset. However, the evidence remains limited by the retrospective design, risk of selection bias, exclusion of important confounding populations such as patients with HIV or autoimmune disease, and the absence of true external validation. In addition, although the model performed well statistically, its applicability outside the original study setting remains uncertain. Overall, the quality of evidence is Low.

#### Recommendation for Integrating Into Clinical Practice

Based on GRADE methodology, we conditionally recommend this multivariable laboratory prediction model as an adjunctive early screening tool in patients with suspected spinal tuberculosis when definitive microbiological testing is delayed or unavailable. Clinicians should not use isolated hematologic markers alone to diagnose STB, and the model should not replace biopsy, molecular testing, or appropriate imaging. Its greatest potential value is in raising or lowering early clinical suspicion in resource-constrained settings while definitive diagnostic work-up is ongoing.

### Surgical Approach Selection in Thoracolumbar Spinal Tuberculosis

**Article 3: Tang Y, Wu WJ, Yang S, et al.** *Surgical treatment of thoracolumbar spinal tuberculosis—a multicentre, retrospective, case-control study.* **J Orthop Surg Res. 2019.**

#### Clinical Rationale

Once the indication for surgery in thoracolumbar spinal tuberculosis has been established, the next practical question is which surgical approach offers the best balance between adequate debridement, neural decompression, deformity correction, stabilization, and perioperative morbidity.^12^ The main options in routine practice are an anterior-only approach, a combined anterior-posterior approach, and a posterior-only approach.^13^ Each has theoretical advantages and disadvantages: anterior surgery allows direct access to the diseased vertebral body, combined surgery may offer greater reconstructive stability in more destructive lesions, and posterior-only surgery may reduce operative trauma while still permitting decompression, correction, and fixation in selected patients. Tang et al was selected because it addresses exactly this decision point in a multicentre comparative cohort and therefore helps inform how surgeons may choose between standard operative approaches in thoracolumbar spinal tuberculosis.^14^

#### Clinical Summary

Tang et al performed a multicentre retrospective case-control study of 132 patients with thoracolumbar spinal tuberculosis treated across six hospitals. Patients underwent one of three surgical strategies: anterior-only, posterior-only, or combined anterior-posterior surgery. The three surgical groups comprised 22 patients treated by anterior-only approach, 79 by combined anterior-posterior approach, and 31 by posterior-only approach across six institutions. Across groups, the study assessed clinical efficacy and safety, including pain, neurologic recovery, fusion, deformity correction, and perioperative parameters. All three approaches were effective in achieving infection control and clinical recovery, but the posterior-only approach was associated with shorter operative time, less blood loss, and better maintenance of kyphosis correction than the anterior-only approach, while still achieving satisfactory fusion and neurologic improvement. The study therefore suggests that, in appropriately selected thoracolumbar STB cases requiring surgery, posterior-only surgery may provide equivalent disease control with lower perioperative burden than more invasive alternatives.

#### Methodological Review

This study has several strengths for a surgical STB paper: it is **multicenter**, includes a **reasonable sample size for a relatively uncommon condition**, and compares **standard surgical approaches** that are directly relevant to daily spine practice. These features make it editorially stronger than highly niche single-center technical series. However, the evidence remains limited by its **retrospective design**, probable **selection bias in assignment of surgical approach**, and the absence of randomization. Because operative approach selection in spinal TB is usually influenced by anatomy, deformity severity, neurologic status, surgeon preference, and institutional expertise, confounding by indication is likely. Accordingly, the study supports comparative inference, but not a definitive causal conclusion that one approach is universally superior. Overall, the quality of evidence is **Low**.

#### Recommendation for Integrating Into Clinical Practice

**Based on GRADE methodology, we conditionally recommend a posterior-only surgical approach as the preferred option for many patients with thoracolumbar spinal tuberculosis requiring operative treatment, provided that adequate debridement, decompression, deformity correction, and stabilization can be achieved through that approach.** Surgery is indicated in a minority of STB patients; the majority of uncomplicated cases are managed effectively with antitubercular chemotherapy alone. When surgery is required, posterior-only approaches are favoured in most cases, with anterior-only and combined approaches reserved for selected complex anatomical scenarios. Anterior-only and combined anterior-posterior approaches remain appropriate in selected cases depending on lesion location, extent of vertebral destruction, abscess burden, sagittal deformity, and surgeon expertise. This recommendation is conditional because the supporting evidence is retrospective and approach selection is strongly influenced by case complexity and local practice patterns.

### Defining Imaging-Based Healed Status in Spinal Tuberculosis

Article 4: Mittal S, Jain AK, Chakraborti KL, Aggarwal AN, Upreti L, Bhayana H. Evaluation of Healed Status in Tuberculosis of Spine by Fluorodeoxyglucose-positron Emission Tomography/Computed Tomography and Contrast Magnetic Resonance Imaging. Indian J Orthop. 2019;53:160-8.

#### Clinical Rationale

Once clinical improvement has occurred in spinal tuberculosis, the next major challenge is deciding when antitubercular therapy can be stopped safely. This decision is difficult because pain, laboratory markers, plain radiographs, and even MRI findings may improve at different rates, and persistent contrast enhancement on MRI does not always indicate ongoing active infection.^15^ Mittal et al was selected because it specifically addresses this practical question: how imaging can be used to define healed status in spinal TB and thereby guide the end-point of treatment in patients whose response remains uncertain on conventional follow-up.^16^

#### Clinical Summary

Mittal et al prospectively evaluated 37 patients with spinal tuberculosis using contrast MRI and FDG-PET/CT to determine healed status and guide discontinuation of antitubercular therapy. Patients were reassessed beginning at 9 months, and ATT was stopped when healing was demonstrated on contrast MRI or when FDG-PET/CT showed no metabolic activity. Healed status on contrast MRI was defined as complete resolution of pre- and paravertebral abscess, replacement of marrow edema by fat or calcification on T1-and T2-weighted sequences, and absence of contrast enhancement. A resolving but active lesion was defined as decreased abscess size with partial fatty replacement of marrow and persistent contrast enhancement. On FDG-PET/CT, healed status was defined as complete absence of FDG uptake, with a standardised uptake value of zero in both bone and soft tissue. Twenty-eight patients reached healed status, but the duration of treatment varied widely, from 9 to 48 months. Among healed cases, 11 were judged healed by both contrast MRI and FDG-PET/CT, 6 had persistent MRI enhancement but no FDG uptake, and 9 were declared healed by FDG-PET/CT when MRI could not be performed, including 6 patients with stainless steel implants. FDG-PET/CT identified healed bone lesions in all healed cases, whereas MRI demonstrated healed response in a smaller proportion of evaluable patients. These findings suggest that metabolic imaging may be particularly useful when MRI remains equivocal or technically limited and support an individualized rather than fixed-duration approach to treatment completion.

#### Methodological Review

This study is notable because it is prospective and directly addresses a clinically important but poorly defined problem: imaging-based confirmation of healed spinal TB. It also uses a structured follow-up strategy and correlates MRI findings with metabolic activity on FDG-PET/CT. However, the evidence remains limited by the small sample size, single-center design, and the fact that serial advanced imaging was provided free of cost within a research setting, which limits external applicability. In addition, the study does not establish that FDG-PET/CT should be used routinely in all patients but rather suggests value in selected equivocal cases. Overall, the quality of evidence is **Low**.

#### Recommendation for Integrating Into Clinical Practice

**Based on GRADE methodology, we conditionally recommend FDG-PET/CT as an adjunctive tool to define healed status in selected patients with spinal tuberculosis when the end-point of treatment remains uncertain on clinical assessment and contrast MRI, or when MRI follow-up is limited by metallic implants.** Imaging-based healed status reflects a clinicoradiological treatment completion endpoint and does not constitute proof of bacteriological eradication. Persistent contrast enhancement on MRI alone should not automatically be interpreted as ongoing active infection. FDG-PET/CT may be most useful when the key clinical question is whether ATT can be safely discontinued, but its broader routine use is limited by cost, access, and the low-certainty evidence base.

## Discussion

A summary of the four reviewed studies, including study design, key findings, methodological limitations, GRADE evidence level, and recommendation strength, is presented in Table 1. The present review highlights that the most consequential uncertainties in spinal tuberculosis are not confined to one phase of care but extend from early diagnostic suspicion to treatment completion. The selected studies suggest that management of STB is best understood as a sequence of distinct clinical decision points: identifying probable disease early, refining diagnosis when standard investigations are insufficient, selecting an appropriate operative strategy when surgery is indicated, and determining when biological healing has occurred. Considered together, these studies support an individualized and multimodal approach to care, while also underscoring an important and somewhat unexpected observation: despite the persistent global burden of spinal tuberculosis, the evidence base supporting many key clinical decisions remains limited and is dominated by low-certainty studies.

**Table 1.** Summary of Reviewed Studies With GRADE Evidence Assessment

Study	Design	Sample	Key findings	Main limitations	Recommendation	GRADE evidence level	Recommendation strength
Altini et al. J Clin Med. 2020 Comparison of MRI and FDG-PET/CT in diagnosis of spondylodiscitis	Retrospective diagnostic cohort	n = 56 (18 with STB)	MRI: sensitivity 100%, specificity 60%, accuracy 97%. FDG-PET/CT: sensitivity 92%, specificity 100%, accuracy 94%. In the STB subgroup (n=18), modalities were concordant in 16/18 patients. FDG-PET/CT showed higher specificity; MRI showed higher sensitivity.	Retrospective design. Small sample size. Indirectness: mixed spondylodiscitis cohort, not STB-specific. Results not directly generalisable to STB alone.	Conditionally recommend FDG-PET/CT as adjunctive imaging when MRI is inconclusive, contraindicated, or limited by artifact. MRI remains first-line. FDG-PET/CT does not establish a diagnosis of STB and does not replace microbiological confirmation.	Low	Conditional
Hu et al. Front Cell Infect Microbiol. 2023 Predictive model for early diagnosis of STB based on conventional laboratory indices	Retrospective multicenter cohort with internal validation	n = 206 (105 STB, 101 non-STB)	Seven-variable nomogram (TB antibody, IGRAs, RBC, Mono%, RDW, AST, BUN) developed by LASSO + logistic regression. AUC 0.947 (training), 0.919 (validation). Individual markers performed poorly; combined model markedly improved discrimination. Total nomogram score >400 corresponds to predicted STB probability >0.5.	Retrospective design. Risk of selection bias. No external validation cohort. Excludes HIV-positive patients and those with autoimmune disease. Generalisability outside original setting uncertain.	Conditionally recommend as adjunctive early screening tool when definitive microbiological testing is delayed or unavailable. Should not replace biopsy, molecular testing, or imaging. Greatest utility in resource-constrained settings.	Low	Conditional
Tang et al. J Orthop Surg Res. 2019 Surgical treatment of thoracolumbar STB — multicentre retrospective case-control study	Retrospective multicentre case-control study	n = 132 (Anterior-only: 22 Combined: 79 Posterior-only: 31)	All three approaches achieved bone fusion, pain relief, and neurological recovery. Posterior-only approach: shorter operative time (257 min) and less blood loss (806 ml) vs combined approach (423 min, 1187 ml). Kyphosis angle loss at final follow-up: anterior-only 5.45°, combined 1.82°, posterior-only 2.21°. Posterior-only and combined superior to anterior-only in kyphosis maintenance.	Retrospective design. Probable selection bias in surgical approach assignment. No randomisation. Strong confounding by indication (lesion severity, deformity, surgeon preference). Relatively short mean follow-up (29 months).	Conditionally recommend posterior-only approach as preferred surgical strategy in most patients with thoracolumbar STB requiring operative treatment, provided adequate debridement, decompression, deformity correction, and stabilisation can be achieved. Anterior and combined approaches remain appropriate in selected cases.	Low	Conditional
Mittal et al. Indian J Orthop. 2019 Evaluation of healed status in STB by FDG-PET/CT and contrast MRI	Prospective single-centre cohort	n = 37 (28 achieved healed status)	Healed status on MRI defined as: complete resolution of abscess, marrow edema replaced by fat/calcification, absence of contrast enhancement. Healed status on FDG-PET/CT defined as: SUV = 0 in bone and soft tissue. FDG-PET/CT demonstrated healed bone lesion in 28/28 (100%) vs MRI in 13/19 (68%) evaluable patients. In 6 patients with stainless steel implants, FDG-PET/CT was the only modality able to confirm healed status. Time to healed status ranged from 9 to 48 months.	Small sample size. Single-centre design. Advanced imaging provided free of cost within research setting, limiting external applicability. Persistent MRI contrast enhancement in healed cases may reflect repair rather than active disease.	Conditionally recommend FDG-PET/CT as adjunctive tool to define treatment completion endpoint when clinical assessment and contrast MRI leave uncertainty, or when MRI is limited by metallic implants. Persistent MRI enhancement alone should not be interpreted as ongoing active infection. Imaging-based healed status reflects a clinicoradiological endpoint and does not constitute proof of bacteriological eradication.	Low	Conditional

STB = spinal tuberculosis; MRI = magnetic resonance imaging; FDG-PET/CT = fluorodeoxyglucose positron emission tomography/computed tomography; ATT = antitubercular therapy; AUC = area under the curve; SUV = standardised uptake value; IGRAs = interferon-gamma release assays; RBC = red blood cell count; Mono% = monocyte percentage; RDW = red cell distribution width; AST = aspartate aminotransferase; BUN = blood urea nitrogen. GRADE evidence levels: High, Moderate, Low, Very Low. Recommendation strength: Strong or Conditional.

A major theme emerging from the reviewed literature is the central but incomplete role of imaging. MRI remains the cornerstone of evaluation in suspected STB because of its ability to define the anatomical extent of vertebral destruction, epidural extension, abscess formation, neural compression, and deformity. However, both the Altini and Mittal studies illustrate that structural imaging alone does not resolve every clinically relevant question. In the diagnostic setting, MRI may be limited by artifact, contraindication, or reduced specificity in the presence of advanced degenerative changes. In the follow-up setting, persistent contrast enhancement may reflect ongoing reparative inflammatory activity rather than active infection. These observations support a more selective use of FDG-PET/CT, not as a replacement for MRI, but as an adjunct in specific situations where standard imaging leaves clinically relevant uncertainty. This interpretation is also consistent with the broader spinal infection literature, in which metabolic imaging has shown promise in differentiating active disease from residual structural abnormality, but has not yet been incorporated into a universally applicable STB algorithm.

The study by Hu et al addresses a different but equally important problem: the gap between clinical suspicion and microbiological confirmation. In many patients, biopsy, culture, histopathology, or molecular diagnostics are either delayed or unavailable at the point when early therapeutic decisions must be made. A laboratory-based multivariable model derived from routine indices is therefore attractive because it reflects a realistic clinical need, particularly in resource-limited settings. At the same time, its role should be interpreted cautiously. The model improves early discrimination, but does not establish a definitive diagnosis and should not displace tissue-based confirmation where feasible. Its principal value lies in raising suspicion earlier and in helping clinicians prioritize further diagnostic work-up in patients whose presentation overlaps with pyogenic infection, brucellosis, or spinal neoplasia. In this regard, the study is best viewed as an adjunct to clinical reasoning rather than a stand-alone diagnostic solution.

The surgical domain is particularly noteworthy because it reveals a mismatch between disease burden and evidentiary maturity. Spinal tuberculosis remains a common indication for operative intervention in many regions of the world, especially in the presence of neurological deficit, progressive deformity, instability, or extensive abscess formation. Yet, high-quality comparative surgical studies remain relatively scarce, and much of the literature continues to rely on retrospective cohort data. This is somewhat surprising given the incidence and severity of surgically relevant STB. Within this context, the study by Tang et al is valuable because it addresses a practical question that surgeons face after the decision to operate has already been made: which surgical approach offers the most favorable balance between debridement, decompression, deformity correction, stabilization, and perioperative morbidity in thoracolumbar STB. Their findings suggest that posterior-only surgery may provide comparable clinical and radiographic outcomes with lower operative burden in many cases. However, even this conclusion should remain conditional. Selection of surgical approach in spinal tuberculosis is inevitably influenced by lesion location, extent of anterior column destruction, abscess burden, sagittal deformity, neurological compression, and surgeon experience. Accordingly, the most defensible interpretation of the current literature is not that one approach is universally superior, but that posterior-only surgery appears to be an effective default strategy in many thoracolumbar cases, while anterior-only and combined approaches remain important in selected anatomical situations.

Another important issue concerns the definition of healed status and the duration of antitubercular therapy. In contrast to pulmonary tuberculosis, spinal tuberculosis lacks a simple microbiological marker that clearly defines treatment completion. This has contributed to major variation in treatment duration and to persistent disagreement regarding the optimal endpoint of therapy. The Mittal study is therefore particularly relevant because it prospectively examined healing using both contrast MRI and FDG-PET/CT and demonstrated that the time required to achieve imaging-defined healed status varies considerably between patients. The study also showed that persistent MRI enhancement is not synonymous with ongoing disease activity and that FDG-PET/CT may be especially useful when MRI remains equivocal or cannot be interpreted because of metallic instrumentation. These findings support the concept that treatment duration in STB should not be determined solely by a fixed chronological threshold. At the same time, the practical implications must be interpreted within the limitations of the study, including its small sample size, single-center design, and the fact that advanced imaging was made available in a research setting without cost to the patient. FDG-PET/CT is therefore best considered a selective problem-solving tool in complex follow-up scenarios rather than a universal standard for monitoring. Avoidance of unnecessarily prolonged antitubercular therapy is an important clinical goal, given the risks of drug toxicity, poor compliance, and emergence of resistance associated with extended treatment courses.

Across all four domains, feasibility remains inseparable from interpretation of the evidence. Some of the reviewed tools, particularly FDG-PET/CT, may offer important additional information but are not available in many environments where spinal tuberculosis is most prevalent. Conversely, MRI and routine laboratory indices are more widely accessible and therefore more likely to shape day-to-day practice. This tension between diagnostic sophistication and global applicability is central to STB care. It also explains why many recommendations in this review remain conditional: they are shaped not only by methodological limitations, but also by variation in access to imaging, laboratory testing, surgical expertise, and longitudinal follow-up.

Perhaps the most important general observation is that the overall study quality remains lower than expected for a disease with such substantial clinical impact. Three of the four selected studies are retrospective, and even the strongest prospective work remains limited by sample size and setting. This is especially striking in surgical TB research, where major treatment decisions continue to rely predominantly on retrospective institutional experience despite the frequency with which surgeons must manage instability, deformity, and neurological compromise. The field would benefit substantially from prospective multicentre studies with standardized definitions of diagnosis, healing, surgical indications, and outcome assessment. In particular, validation of laboratory triage tools, comparative evaluation of surgical approaches in defined anatomical subgroups, and more reproducible criteria for treatment completion should be considered priorities for future research. The recommendations derived from this review are broadly consistent with WHO guidance, which supports MRI as the preferred imaging modality for suspected spinal tuberculosis, endorses standard four-drug ATT regimens, and does not prescribe a fixed treatment duration for spinal TB — an approach consistent with the individualised imaging-guided endpoint advocated by Mittal et al. The laboratory prediction model and surgical approach recommendations are not directly addressed by WHO guidelines but align with the principle of individualised, resource-appropriate clinical decision-making. None of the four reviewed studies specifically included patients with drug-resistant spinal tuberculosis. The recommendations derived from this review therefore cannot be directly extrapolated to this population, which represents an important gap in the current evidence base and a priority area for future research.

In summary, the current literature supports a pragmatic but cautious approach to spinal tuberculosis. MRI remains the first-line imaging modality, while FDG-PET/CT appears most useful in selected cases of diagnostic or follow-up uncertainty. Laboratory-based multivariable models may improve early suspicion, especially when definitive testing is delayed, but do not replace tissue diagnosis. When surgery is indicated in thoracolumbar disease, posterior-only approaches appear attractive in many patients, although operative planning must remain individualized. Finally, treatment completion should be guided by biological and imaging response rather than elapsed time alone whenever uncertainty persists. These conclusions are clinically relevant, but they also reflect the limitations of the available evidence and the continuing need for more rigorous studies in this field.
